# Am80 (tamibarotene) and ATRA induce highly similar molecular responses and myeloid differentiation in non-APL AML, enhanced by LSD1/GCN5 inhibition and increased RARA expression

**DOI:** 10.1186/s12885-026-16388-2

**Published:** 2026-06-27

**Authors:** Faezeh Ghazvini Zadegan, Clara Stanko, Franziska Fiedler, Laura Ölsner, Setenay Gupse Özcan, Jacqueline Schütt, Tina M. Schnöder, Yordan Sbirkov, Lukasz Szymanski, Sven Stengel, Peter Dittrich, Jörg P. Müller, Florian H. Heidel, Andreas Hochhaus, Sebastian Scholl, Ulf Schnetzke, Annamaria Brioli, Tino Schenk

**Affiliations:** 1 Klinik für Innere Medizin II, Jena University Hospital, Comprehensive Cancer Center Central Germany, Jena, Germany; 2 Institute of Molecular Cell Biology, Jena University Hospital, Jena, Germany; 3 Department of Mathematics and Computer Science, Friedrich Schiller University Jena, Jena, Germany; 4 Hematology, Hemostasis, Oncology and Stem Cell Transplantation, Hannover Medical School (MHH), Hannover, Germany; 5 Department of Internal Medicine C, University Medicine Greifswald, Greifswald, Germany; 6 Medical University of Plovdiv, Plovdiv, Bulgaria; 7 Research Institute at Medical University of Plovdiv, Plovdiv, Bulgaria; 8 Department of Molecular Biology, Institute of Genetics and Animal Biotechnology, Polish Academy of Science, Magdalenka, Poland; 9 Department of Neuropediatrics, Jena University Hospital, Jena, Germany; 10 Leibniz Institute on Aging, Fritz-Lipmann-Institute, Jena, Germany; 11 Cellular Therapy Center (CTC), Hannover Medical School (MHH), Hannover, Germany

**Keywords:** Am80, Tamibarotene, ATRA, RARA, AML

## Abstract

**Background:**

Acute myeloid leukemia (AML) remains a challenging disease with a poor prognosis, necessitating more personalized therapeutic strategies. Retinoic acid receptor (RAR) activation is crucial for myeloid differentiation, but non-APL AML cells resist differentiation induced by the pan-RAR agonist all-trans retinoic acid (ATRA). Am80 (tamibarotene), a specific RARA agonist, has been reported to partially overcome this resistance in AML with high RARA expression. However, its effects on myeloid differentiation, especially in comparison to ATRA, remain understudied.

**Methods:**

In this study we compared the effects of Am80 and ATRA in non-APL AML samples, focusing on subsets with high RARA and/or RARG expression, using molecular and phenotypic differentiation assessments.

**Results:**

In contrast to previous findings, Am80 showed no advantage over ATRA in inducing differentiation in AML cell lines or primary samples, regardless of RARA and RARG expression levels. Cotreatment with inhibitors of the epigenetic modifiers LSD1 and GCN5, which facilitates retinoid-induced myeloid differentiation, enhanced the effects of both Am80 and ATRA to the same extent, with more pronounced responses observed in RARA-high samples. Gene expression analysis revealed identical molecular responses to Am80 and ATRA.

**Conclusions:**

The study provides evidence that ATRA can be substituted by the more stable Am80 in retinoid-based AML therapies. It also identifies elevated RARA expression as a potential marker for sensitivity to combination therapy with retinoids and epigenetic inhibitors in AML.

**Supplementary Information:**

The online version contains supplementary material available at 10.1186/s12885-026-16388-2.

## Background

Acute myeloid leukemia (AML) is characterized by a blockade in myeloid differentiation, which is critically regulated by retinoic acid receptor (RAR) transcription factors. There are three RAR isotypes, RARA, RARB, and RARG, which heterodimerize with retinoid X receptors (RXRA, RXRB, and RXRG) and bind to retinoic acid response elements to control transcriptional programs required for myeloid maturation. Upon ligand binding, these receptors exchange coregulators and activate genes necessary for differentiation. Acute promyelocytic leukemia (APL), driven by the PML-RARA fusion, can be effectively targeted by all-trans retinoic acid (ATRA), whereas other AML subtypes, despite expressing functional RARA, are generally resistant to ATRA-induced differentiation [1].

While RARA is required for the induction of granulocytic differentiation, RARG plays a central role in maintaining the self-renewal of hematopoietic stem cells (HSCs) [2]. To prevent activation of RARG by ATRA, specific RARA-agonists, such as Am80 (SY-1425, tamibarotene) have been developed.

McKeown and colleagues (2017, Cancer Discovery) reported Am80 to be specifically active in a subset of RARA-overexpressing AML that carry a particularly strong superenhancer (SE) at the RARA gene locus. In contrast to ATRA, Am80 was found to be 1- to 21-fold more potent in reducing the growth of AML cell lines and cells of a Patient-Derived Xenograft (PDX) model with high RARA expression. In their setting, Am80 induced myeloid differentiation in RARA-high, but not RARA-low, cell lines and ex vivo treated primary samples [3]. In a recent clinical trial, AML patients with high levels of RARA responded better to treatment with Am80 in combination with azacytidine [4]. Am80 has already been approved for the treatment of APL in conjunction with arsenic trioxide (ATO) in Japan and has been granted a fast-track designation for high-risk myelodysplastic syndrome by the FDA in 2023 [5].

Besides receptor expression, epigenetic mechanisms also contribute to retinoid resistance in non-APL AML. In particular, LSD1 (KDM1A) and GCN5 (KAT2A) have been implicated in maintenance of the differentiation block, and inhibition of these factors has been shown to enhance ATRA responsiveness [6, 7]. These observations suggest that limited execution of retinoid-driven transcriptional programs may constrain differentiation even when RARA is expressed.

In this study, we investigated whether the RARA-selective agonist Am80 can overcome the differentiation block in non-APL AML compared with the pan-RAR agonist ATRA. We examined the effects of both retinoids in 6 AML cell lines and 67 primary AML samples, with particular attention to subsets with high RARA and/or RARG expression. In addition, we tested whether inhibition of LSD1 and GCN5 enhances responses to ATRA and Am80, and whether these effects are associated with receptor expression state.

## Methods

### Cell lines, primary cells, and cell culture

Six AML cell lines (Kasumi-1, HL-60, OCI-AML2, THP-1, MV4-11, and OCI-AML3) were purchased from DSMZ, maintained under standard conditions, and treated for 3 days with 0.1 µM ATRA, 0.1 µM Am80, 0.1 µM GSK-LSD1, and 100 µM MB3. Primary AML samples from 67 patients (Supplementary Table 1) were isolated from peripheral blood mononuclear cells using StemSep columns (STEMCELL Technologies, Canada), cultured in Stemline II medium (Sigma, Hamburg, Germany), and treated for 3 days with 1 µM ATRA, 1 µM Am80, 0.1 µM GSK-LSD1, and 100 µM MB3. Use of primary human samples was approved by the respective institutional ethics committees (approval numbers 4753-04/16 and BB 199/20). All samples were obtained with informed consent and used in accordance with the Declaration of Helsinki.

### Cell analyses

Surface marker expression was assessed by flow cytometry using a BD LSRFortessa™. Cells were pre-blocked with Human TruStain FcX to prevent non-specific Fc-receptor binding and stained with anti-CD11b-FITC (BioLegend, #301330) and anti-CD117-BV711 (BioLegend, #313230). Data were analyzed using FlowJo software. For cell viability assays, cells were seeded at a density of 10,000 cells/ml, treated with the indicated drug concentrations, and cell viability was measured using the CellTiter-Glo^®^ luminescent cell viability assay (Promega) according to the manufacturer’s protocol.

### RNA-seq and bioinformatic analysis

For RNA-seq, 67 untreated primary AML samples collected before treatment and OCI-AML3 cells treated for 3 h with the indicated compounds were analyzed. Total RNA was isolated using the ZR RNA MiniPrep Kit (Zymo Research), libraries were prepared using the Illumina TruSeq Stranded mRNA Sample Preparation Kit, library quality was assessed with an Agilent 2100 BioAnalyzer, and sequencing was performed on an Illumina HiSeq2500 in 51-cycle single-end high-output mode.

Raw reads were analyzed using Galaxy (usegalaxy.org), and raw count data were subjected to differential expression analysis with DESeq2 using default settings. Unsupervised hierarchical clustering, PCA, and Pearson correlation analyses were performed using Degust (degust.erc.monash.edu) and Graphpad Prism 10. GO Biological Process enrichment analysis was performed using g: Profiler.

### Publicly available gene expression datasets

Gene expression data for 44 AML cell lines were obtained from the DepMap database [8]. TPM values for protein-coding genes were inferred from RNA-seq data using RSEM and are reported as log2(TPM + 1). Gene expression data for 672 primary AML samples were obtained from cBioPortal [9]. RNA-seq expression values were provided by the source dataset as log2-transformed, CQN-normalized RPKM values.

### Statistics

Data were analyzed using one-way analysis of variance (ANOVA) followed by Tukey’s post hoc test, unless otherwise noted. Statistical significance was set at *P* < 0.05. *P < 0.05, **P < 0.01, ***P < 0.001, ****P < 0.0001. Statistical analyses were performed using the GraphPad Prism software (version 9; GraphPad Software Inc.).

## Results

**Fig. 1:**
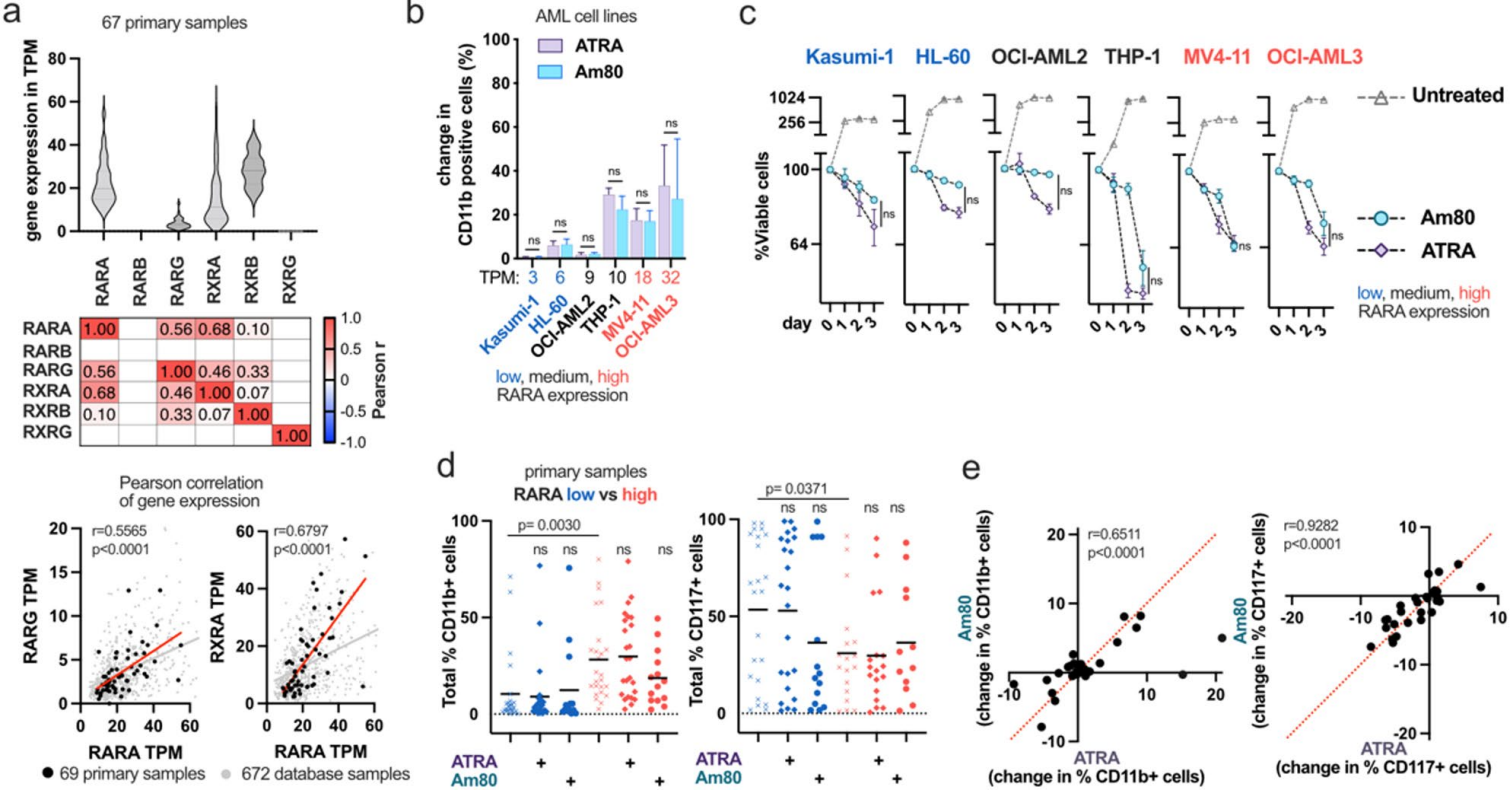
Specific activation of RARA by Am80 (tamibarotene) does not induce greater differentiation than the pan-RAR agonist ATRA in AML. **a** Upper panel: Expression of RAR and RXR family members (TPM) in our cohort of 67 primary AML samples. Middle panel: Pearson correlation matrix of RAR and RXR gene expression. Lower panel: Pearson correlations of RARA versus RARG and RARA versus RXRA in our cohort (black) and in the published AML dataset from Tyner et al. (2018) (grey). **b** Flow cytometric analysis of CD11b expression in six AML cell lines grouped according to baseline RARA expression as RARA-low, -medium, or -high (Kasumi-1, 2.9 TPM; HL-60, 5.86 TPM; OCI-AML2, 9.19 TPM; THP-1, 10.43 TPM; MV4-11, 18.34 TPM; OCI-AML3, 32.45 TPM) after 3 days of treatment with ATRA (0.1 µM) or Am80 (0.1 µM). **c** Cell viability of the six AML cell lines after 3 days of treatment, measured by CellTiter-Glo. **d** Flow cytometric analysis of CD11b and CD117 expression in 50 primary AML samples after 3 days of treatment with ATRA (1 µM) or Am80 (1 µM). Samples with RARA TPM < 15 are shown in blue (RARA low), and samples with RARA TPM > 22 are shown in red (RARA high). **e** Pearson correlation of treatment-induced changes in CD11b and CD117 expression in primary AML samples treated with ATRA or Am80

For this study, we analyzed six AML cell lines and 67 primary AML samples. To demonstrate the representativeness of this cohort, we compared their global gene expression profiles with publicly available datasets comprising 44 myeloid cell lines and 672 primary AML samples [10, 11]. Consistently, RARA was the most abundant retinoic acid receptor, with an expression level approximately fivefold higher than that of RARG. A significant correlation was observed between RARA and RARG expression, and both were co-expressed with RXRA. Because LSD1 and GCN5 have previously been implicated in resistance to retinoid-induced differentiation [6, 7], we also examined their expression in the reference datasets and in relation to retinoid response, as rationale for the subsequent combination experiments (Fig. 1a, Suppl. Figure 1a and Suppl. Figure 2a).

To evaluate prior reports suggesting that AML with elevated RARA expression are particularly responsive to selective RARA activation [3, 12], we treated six AML cell lines exhibiting varying RARA levels with either ATRA or Am80 for three days. Notably, we observed no differential effects between the two retinoids, but higher RARA expression correlated with greater induction of the myeloid differentiation marker CD11b and overall retinoid sensitivity (Fig. 1b, c). Supporting, data from 44 myeloid cell lines (depmap.org) indicated increased sensitivity to RAR agonists in cells with higher RARA expression (Suppl. Figure 1b-d). The weak expression of RARG in cell lines did not correlate with treatment response, possibly explaining the lack of efficacy difference between the RARA-specific agonist Am80 and the pan-RAR agonist ATRA.

Primary AML specimens are characterized by higher RARG levels and are known to demonstrate significantly greater resistance to differentiation induction by ATRA, compared to cell lines. Although previous studies suggested that AML is particularly sensitive to specific RARA activation by Am80 [3, 12], our findings demonstrate that Am80 has no greater effect in primary AML, as indicated by upregulation of the myeloid differentiation marker CD11b and downregulation of the immature hematopoietic/progenitor cell marker CD117 in our cohort (Fig. 1d, e and Suppl. Figure 2e). RARA-high samples exhibited elevated baseline CD11b and reduced CD117 expression; however, this did not translate into an increased differentiation response. Consistently, Spearman correlation analysis showed no significant association between baseline CD11b/CD117 expression and treatment-induced changes in these markers in primary AML samples (Fig. 1d and Suppl. Figure 2c). Responses to ATRA and Am80 were highly correlated across treated samples (Fig. 1e).

**Fig. 2:**
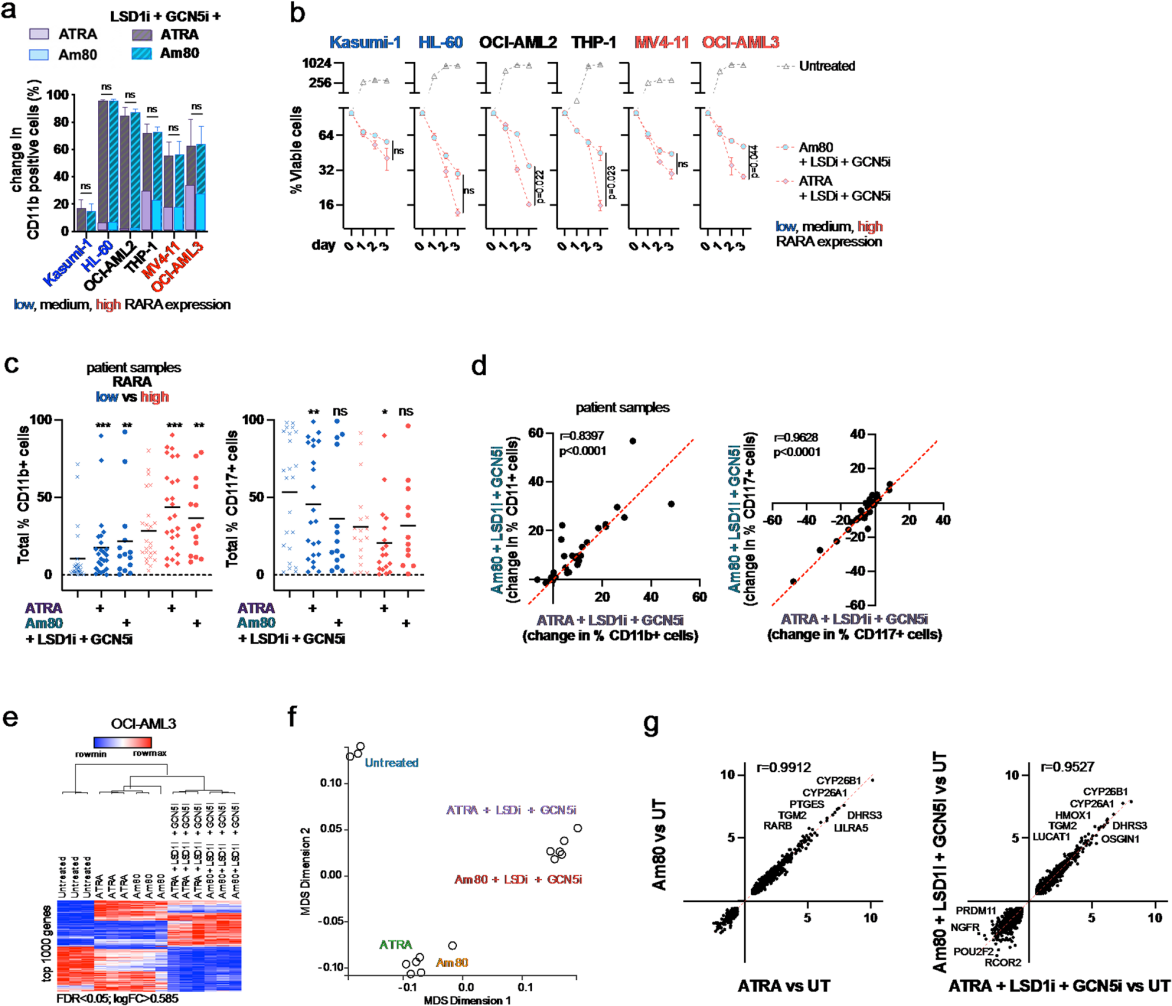
Inhibition of LSD1 and GCN5 enables highly similar responses to Am80 and ATRA, especially in RARA-high AML. **a** Flow cytometric analysis of surface expression of the myeloid differentiation marker CD11b in six AML cell lines categorized as RARA-low, -medium, or -high based on baseline RARA mRNA expression levels (TPM), ranked across the six cell lines (low: Kasumi-1, 2.9 TPM; HL-60, 5.86 TPM; medium: OCI-AML2, 9.19 TPM; THP-1, 10.43 TPM; high: MV4-11, 18.34 TPM; OCI-AML3, 32.45 TPM) after 3 days of treatment with ATRA (0.1µM), Am80 (0.1µM), LSD1 inhibitor (LSD1i) GSK-LSD1 (0.1µM) and the GCN5 inhibitor (GCN5i) MB3 (100µM). Non-dashed columns show response to ATRA or Am80 alone, as shown in Fig. 1b. **b** Growth of the six AML cell lines after treatment was measured for 3 days using the CellTiter-Glo^®^ luminescent cell viability assay (Promega). **c** Flow cytometric analysis of CD11b and CD117 expression in 50 primary AML samples grouped as RARA-high (> 22 TPM) or RARA-low (< 15 TPM), after 3 days of treatment with ATRA (1 µM) or Am80 (1 µM) in combination with GSK-LSD1 (0.1 µM) and MB3 (100 µM), compared with untreated cells. **d** Pearson correlation of the change in surface marker expression following treatments. **e** Heatmap of unsupervised hierarchical clustering of genes in OCI-AML3 cells differentially expressed in response to treatment with combinations with ATRA (0.1µM), Am80 (0.1µM), LSD1 inhibitor (LSD1i) GSK-LSD1 (0.1µM) and the GCN5 inhibitor (GCN5i) MB3 (100µM). A red-blue color scale was used to reflect standardized gene expression, with red indicating higher expression and blue indicating lower expression. **f** Principal Component Analysis of global gene expression in OCI-AML3 following treatment with ATRA (0.1µM), Am80 (0.1µM), LSD1 inhibitor (LSD1i) GSK-LSD1 (0.1µM) and the GCN5 inhibitor (GCN5i) MB3 (100µM). **g** Pearson correlation of changes in the expression of genes following treatments

Since cell lines with high RARA expression levels exhibited greater sensitivity to retinoids, we hypothesized that a similar effect might be observed in primary AML upon alleviation of the epigenetic differentiation block. As previously shown by us and others, inhibition of the lysine demethylase LSD1 (KDM1A) and the histone acetyltransferase GCN5 (KAT2A) enhances ATRA-induced differentiation [6, 7]. To test this hypothesis, we again compared Am80 and ATRA in combination with LSD1 and GCN5 inhibitors. In AML cell lines, both compounds induced comparable levels of myeloid differentiation, as assessed by CD11b expression (Fig. 2a). Dual inhibition of LSD1 and GCN5 effectively relieved the differentiation block in all tested cell lines, leading to robust myeloid differentiation following retinoid treatment, irrespective of RARA expression levels. Responses to ATRA and Am80 were highly comparable; however, viability assays revealed that in some cell lines, ATRA exerted even stronger effects than Am80 (Fig. 2b).

In primary samples, more than half of the specimens showed a significant response to ATRA when combined with epigenetic inhibition, characterized by an increase in CD11b expression and/or a decrease in CD117. Despite some samples having higher RARG levels, response to RARA-specific activation by Am80 yielded again, extremely similar results. (Fig. 2c, d and Suppl. Figure 2d). Notably, after cotreatment with LSD1 and GCN5 inhibitors, a stronger general retinoid response was observed in RARA-high primary AML samples (Fig. 2c and Suppl. Figure 2d, right panel).

To precisely evaluate transcriptional differences induced by pan-RAR activation by ATRA versus RARA-specific activation by Am80, we performed genome-wide RNA-seq in RARA- and RARG-expressing OCI-AML3 cells 3 h after treatment. ATRA and Am80 induced highly similar transcriptional responses, both alone and in combination with LSD1 and GCN5 inhibitors, as shown by correlation analyses at the single-gene and pathway levels (Fig. 2e-g and Suppl. Figure 3a-c). Both retinoids induced myeloid differentiation genes, and GO term analysis of the top regulated genes supported activation of differentiation-associated biological processes together with suppression of stem/progenitor maintenance programs in the combination setting (Suppl. Figure 3c). Consistently, addition of LSD1 and GCN5 inhibitors downregulated stem cell maintenance-associated genes, including POU2F2 (OCT2), RCOR2, PRDM11, HLA-G, and NGFR, in line with the pattern previously described in HL-60 cells [7] (Fig. 2g and Suppl. Figure 3a-c).

## Discussion

Our study demonstrates that the synthetic retinoid Am80, which selectively targets retinoic acid receptor alpha (RARA), induces molecular and phenotypic differentiation responses equivalent to those of the widely used pan-RAR agonist all-trans retinoic acid (ATRA). While increased RARA levels and retinoid activation by ATRA or Am80 alone are sufficient in cell line models, they appear insufficient to consistently induce robust myeloid differentiation across primary AML samples, contrary to previous reports suggesting an enhanced response of RARA-high AML to Am80 [3]. This lack of response is likely due to downstream blockade of RARA target gene activation by independent mechanisms, including epigenetic repression [6, 7]. Indeed, enabling myeloid differentiation through cotreatment with LSD1 and GCN5 inhibitors, as previously described [6, 7], effectively unblocked the differentiation response to both ATRA and Am80. Again, both compounds induced comparable increases in CD11b expression and decreases in CD117 levels, indicating that the coactivation of retinoic acid receptor gamma (RARG) by ATRA does not exert significant inhibitory effects. Consequently, given its enhanced properties, such as improved stability, Am80 may be considered a viable substitute for ATRA in clinical applications. Another finding was that baseline CD11b/CD117 expression, used here as an immunophenotypic surrogate of differentiation stage, did not significantly correlate with treatment response, suggesting that retinoid sensitivity is not simply determined by the apparent maturation stage of the leukemic blasts. The treatment of non-APL AML with ATRA in combination with LSD1 inhibitors has been clinically tested with some success, but the underlying molecular features remain elusive [13–15].

Here we find that primary AML samples with high RARA expression showed a stronger overall response in the combination setting with LSD1 and GCN5 inhibition, whereas RARA expression alone did not predict a greater differentiation response to single-agent ATRA or Am80.

It is therefore of primary importance to identify those patients who could benefit from such treatment in order to reduce toxicities and rationalize resources. Surrogate surface markers, such as CD120b and CD86, whose expression strongly correlates with high RARA and low LSD1 and GCN5 expression, could be potentially used to identify patients benefitting most from differentiation therapy (Suppl. Figure 2a, b). A better-defined target group of AML or high-risk MDS, together with the advantages of the combination of synthetic retinoids, next-generation LSD1, and the potential addition of GCN5 inhibitors, would likely result in significantly improved responses and potentially longer survival especially in elderly patients.

## Supplementary Information

Supplementary Material 1.

## Data Availability

Data discussed in this publication were deposited in NCBI’s Gene Expression Omnibus and are accessible through GEO Series accession number GSE256476 (Reviewer Token: cvcxccuetfwfvkh) and GSE260773 (Reviewer Token: qviloykqnvcdncb).

## Abbreviations

AML: Acute Myeloid Leukemia
APL: Acute Promyelocytic Leukemia
ATRA: All–Trans Retinoic Acid
Am80: Tamibarotene (RARA–specific agonist)
RARA: Retinoic Acid Receptor Alpha
RARG: Retinoic Acid Receptor Gamma
LSD1 (KDM1A): Lysine–Specific Demethylase 1 A
GCN5 (KAT2A): Lysine Acetyltransferase 2 A

## References

[1] Schenk T, Stengel S, Zelent A. Unlocking the potential of retinoic acid in anticancer therapy. Br J Cancer. 2014;111(11):2039–45. 25412233 10.1038/bjc.2014.412 PMC4260020

[2] Conserva MR, Redavid I, Anelli L, Zagaria A, Specchia G, Albano F. RARG Gene Dysregulation in Acute Myeloid Leukemia. Front Mol Biosci. 2019;6:114. 31709264 10.3389/fmolb.2019.00114 PMC6822255

[3] McKeown MR, Corces MR, Eaton ML, Fiore C, Lee E, Lopez JT, et al. Superenhancer Analysis Defines Novel Epigenomic Subtypes of Non-APL AML, Including an RARalpha Dependency Targetable by SY-1425, a Potent and Selective RARalpha Agonist. Cancer Discov. 2017;7(10):1136–53. 28729405 10.1158/2159-8290.CD-17-0399 PMC5962349

[4] de Botton S, Cluzeau T, Vigil C, Cook RJ, Rousselot P, Rizzieri DA, et al. Targeting RARA overexpression with tamibarotene, a potent and selective RARalpha agonist, is a novel approach in AML. Blood Adv. 2023;7(9):1858–70. 36477975 10.1182/bloodadvances.2022008806 PMC10165187

[5] Tamibarotene Plus Venetoclax/Azacitidine. in Participants With Newly Diagnosed AML. https://clinicaltrialsgov/study/NCT04905407.

[6] Schenk T, Chen WC, Gollner S, Howell L, Jin L, Hebestreit K, et al. Inhibition of the LSD1 (KDM1A) demethylase reactivates the all-trans-retinoic acid differentiation pathway in acute myeloid leukemia. Nat Med. 2012;18(4):605–11. 22406747 10.1038/nm.2661 PMC3539284

[7] Kahl M, Brioli A, Bens M, Perner F, Kresinsky A, Schnetzke U, et al. The acetyltransferase GCN5 maintains ATRA-resistance in non-APL AML. Leukemia. 2019;33(11):2628–39. 31576004 10.1038/s41375-019-0581-y

[8] Tsherniak A, Vazquez F, Montgomery PG, Weir BA, Kryukov G, Cowley GS, et al. Defining Cancer Dependency Map Cell. 2017;170(3):564–76. e16. 28753430 10.1016/j.cell.2017.06.010 PMC5667678

[9] Tyner JW, Tognon CE, Bottomly D, Wilmot B, Kurtz SE, Savage SL, et al. Functional genomic landscape of acute myeloid leukaemia. Nature. 2018;562(7728):526–31. 30333627 10.1038/s41586-018-0623-z PMC6280667

[10] Ghandi M, Huang FW, Jané-Valbuena J, Kryukov GV, Lo CC, Mcdonald ER, et al. Next-generation characterization of the Cancer Cell Line Encyclopedia. Nature. 2019;569(7757):503–8. 31068700 10.1038/s41586-019-1186-3 PMC6697103

[11] Bottomly D, Long N, Schultz AR, Kurtz SE, Tognon CE, Johnson K, et al. Integrative analysis of drug response and clinical outcome in acute myeloid leukemia. Cancer Cell. 2022;40(8):850–64. e9. 35868306 10.1016/j.ccell.2022.07.002 PMC9378589

[12] McKeown MR, Johannessen L, Lee E, Fiore C, di Tomaso E. Antitumor synergy with SY-1425, a selective RARalpha agonist, and hypomethylating agents in retinoic acid receptor pathway activated models of acute myeloid leukemia. Haematologica. 2019;104(4):e138–42. 30337363 10.3324/haematol.2018.192807 PMC6442965

[13] Lübbert M, Schmoor C, Berg T, Kruszewski M, Schittenhelm MM, Götze K, et al. Phase I Study of the LSD1 Inhibitor Tranylcypromine (TCP) in Combination with All-Trans Retinoic Acid (ATRA) and Low-Dose Cytarabine (LDAC) in Elderly, Medically Non-Fit Patients with AML or High-Risk MDS (TRANSATRA trial). Blood. 2022;140(Supplement 1):9087–8.

[14] Tayari MM, Santos HGD, Kwon D, Bradley TJ, Thomassen A, Chen C, et al. Clinical Responsiveness to All-trans Retinoic Acid Is Potentiated by LSD1 Inhibition and Associated with a Quiescent Transcriptome in Myeloid Malignancies. Clin Cancer Res. 2021;27(7):1893–903. 33495312 10.1158/1078-0432.CCR-20-4054 PMC8026558

[15] Wass M, Gollner S, Besenbeck B, Schlenk RF, Mundmann P, Gothert JR, et al. A proof of concept phase I/II pilot trial of LSD1 inhibition by tranylcypromine combined with ATRA in refractory/relapsed AML patients not eligible for intensive therapy. Leukemia. 2021;35(3):701–11. 32561840 10.1038/s41375-020-0892-z PMC7303943

