# Supplementary material for: Am80 (tamibarotene) and ATRA induce highly similar molecular responses and myeloid differentiation in non-APL AML, enhanced by LSD1/GCN5 inhibition and increased RARA expression
Source: BMC Cancer. 2026 Jun 27;26:777. doi: 10.1186/s12885-026-16388-2 (PMC13309949; doi:10.1186/s12885-026-16388-2)
Supplement: Supplementary file 1 — Supplementary Material 1. [file 12885_2026_16388_MOESM1_ESM.docx]

**Am80 (tamibarotene) and ATRA induce highly similar molecular responses and myeloid differentiation in non-APL AML, enhanced by LSD1/GCN5 inhibition and increased RARA expression**

**Supplementary Figures**

- Supplementary Figure 1
- Supplementary Figure 2
- Supplementary Figure 3

**Supplementary Tables**

- Supplementary Table 1: *Patient Characteristics*

## Supplementary Figures


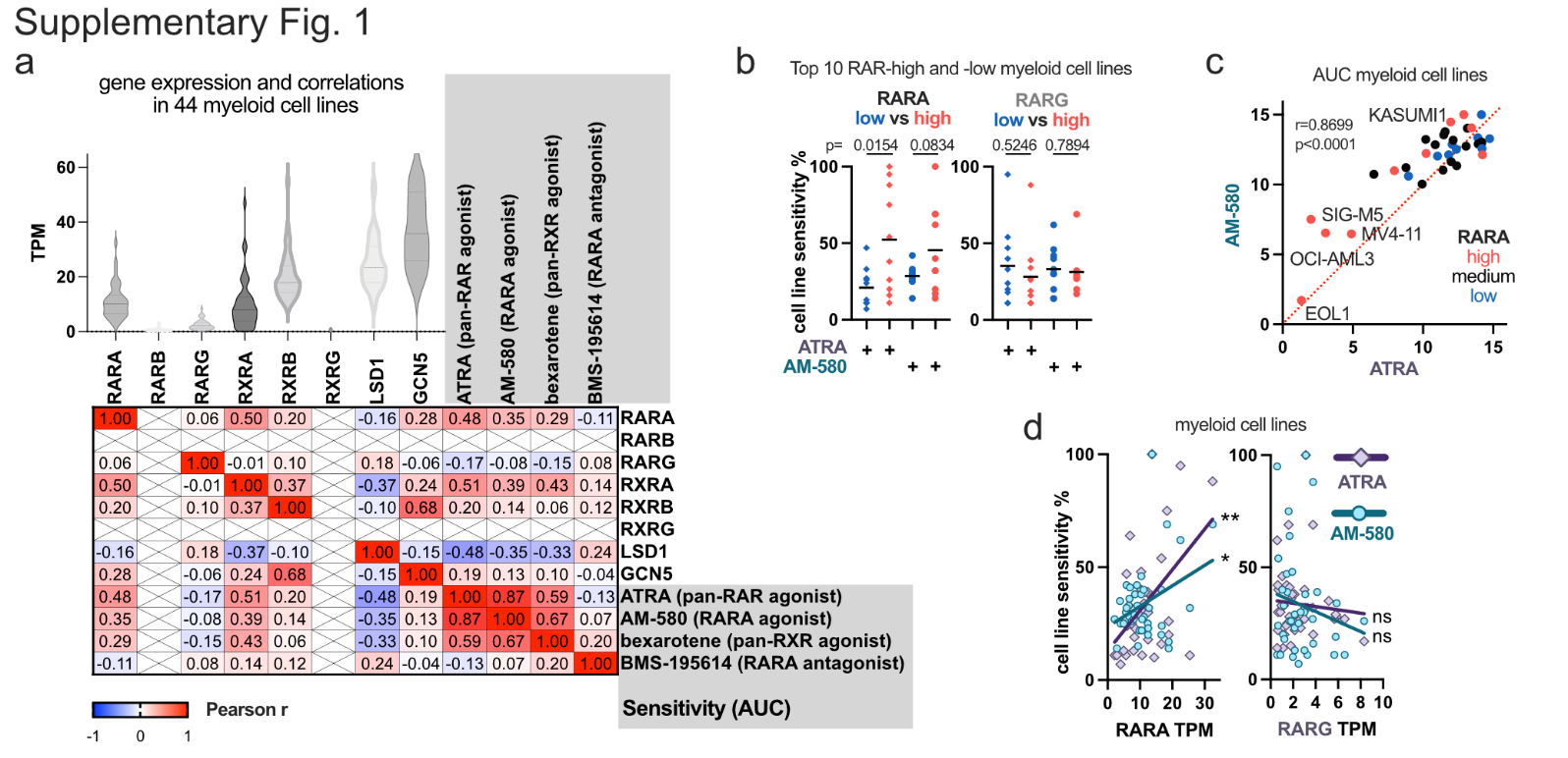


**a** Upper panel: Gene expression levels (TPM) of RAR and RXR family members, as well as LSD1 and GCN5, in 44 myeloid cell lines. Lower panel: Pearson correlation matrix comparing gene expression and drug-sensitivity parameters. Because Am80 was not available in the DepMap dataset, the structurally and functionally related RARA-selective agonist AM-580 was used as a surrogate. In addition, the matrix includes sensitivity data for ATRA, the pan-RXR agonist bexarotene, and the RARA antagonist BMS-195614, based on area-under-the-curve (AUC) values. **b** Sensitivity to retinoids in 10 DepMap myeloid cell lines with high RARA or RARG expression and 10 cell lines with low RARA or RARG expression. High- and low-expression groups were defined by ranking baseline log2(TPM + 1) mRNA expression values and selecting the top 10 and bottom 10 cell lines, respectively. **c** Correlation of drug sensitivity to ATRA and the Am80 surrogate AM-580 across 44 myeloid cell lines using DepMap AUC data; EOL1 was the most sensitive cell line. **d** Pearson correlation of RARA and RARG expression with sensitivity to ATRA and AM-580 in 44 myeloid cell lines using available DepMap AUC data.


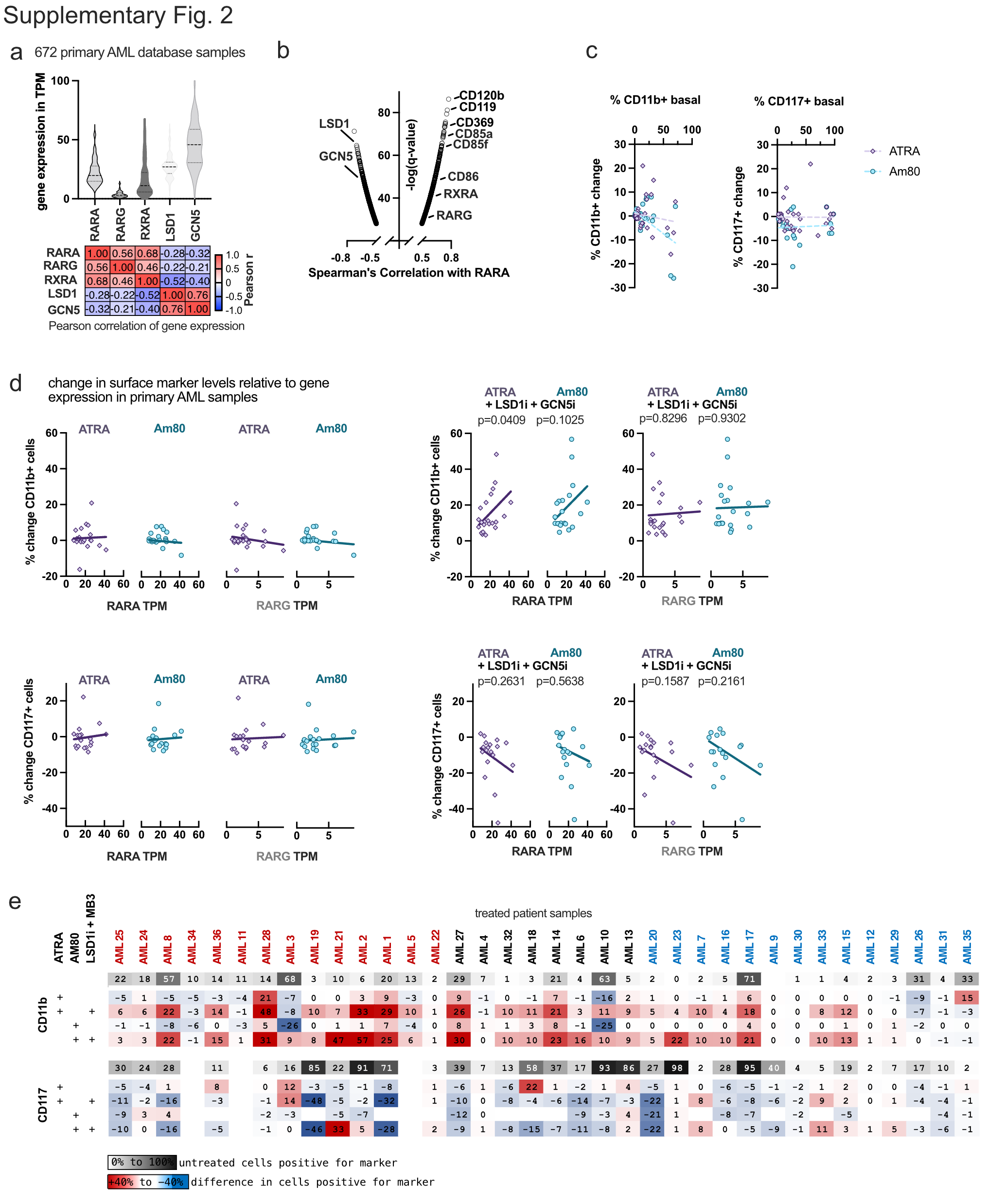


**a** Gene expression (TPM) of RARs and RXRs in 672 primary AML database (cBioPortal) samples and Pearson correlation matrix. **b** Analysis of genes whose expression is significantly positively or negatively correlated (Spearman) with RARA in 672 primary AML. **c** Spearman correlation analysis between baseline CD11b/CD117 expression and treatment-induced changes in primary AML samples treated with 1µM ATRA and 1µM Am80. No significant correlations were observed. Only samples treated with both compounds have been included. **d** Pearson correlation between RARA and RARG gene expression (TPM) and changes in CD11b and CD117 surface marker levels in primary AML samples following 3 days of treatment with ATRA (1 µM) or Am80 (1 µM) alone (left panel) or in combination with the LSD1 inhibitor GSK-LSD1 (0.1 µM) and the GCN5 inhibitor MB3 (100 µM) (right panel). **e** Heatmap of basal (gray) CD11b and CD117 surface marker expression and changes (red to blue) in 35 primary AML samples, following 3 days of treatment with ATRA (1 µM) or Am80 (1 µM) alone and in combination with the LSD1 inhibitor GSK-LSD1 (0.1µM) and the GCN5 inhibitor MB3 (100µM). Samples with a RARA TPM value lower than 15 (RARA low) are labelled blue, samples with a RARA TPM value between 15 and 22 (RARA middle) are labelled black and samples with a RARA TPM value between higher than 22 (RARA high) are labelled black.


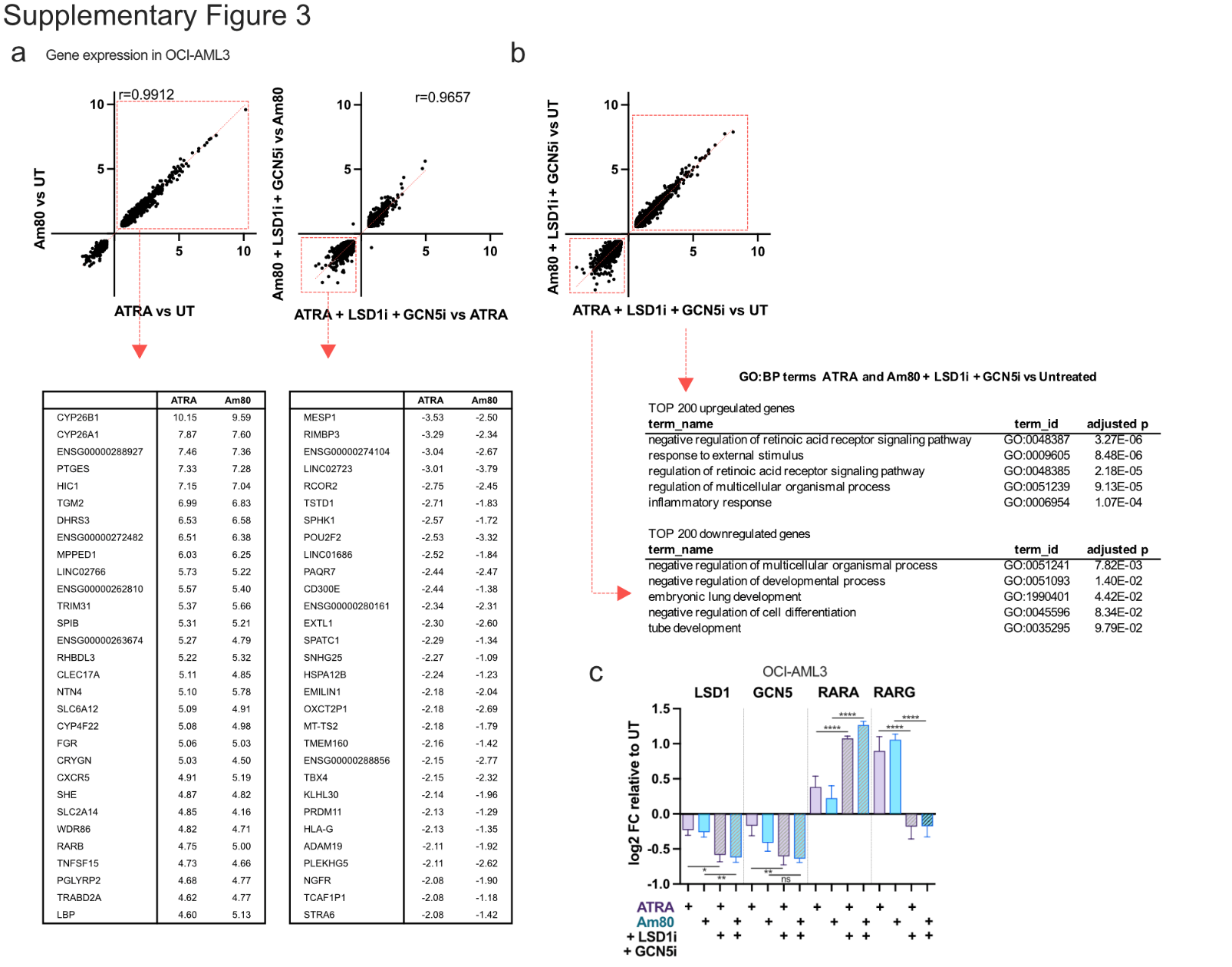


**a** Genes upregulated by ATRA and Am80. **b** Genes downregulated following the addition of the LSD1 and GCN5 inhibitors to ATRA and Am80. **c** Biological Processes (GO terms) enriched in the TOP 200 up or downregulated genes following ATRA or Am80 in combination with LSD1 and GCN5 inhibitors. The analysis was performed using the g:Profiler tool. **c** Gene expression log2 fold-change (log2 FC) 3h following treatment of OCI-AML3 cells as described above.

## Supplementary Tables

***Supplementary Table 1: Patient Characteristics***

| Characteristics | Patients (N=67) |
| --- | --- |
| Median age (range) | 60 years (25 to 91 years) |
| Sex (m/f) | 27/40 |
| FAB (%) |  |
| M0 | 2 (3) |
| M1 | 9 (13) |
| M2 | 8 (12) |
| M4 | 14 (21) |
| M5 | 5 (7) |
| M6 | 1 (1) |
| Not classified | 28 (45) |
| Type (%) |  |
| Primary | 41 (61) |
| Secondary | 14 (21) |
| Relapsed | 11 (16) |
| Therapy-related | 1 (1) |
| Not classified | 0 (0) |
| Blasts in peripheral blood |  |
| >50% | 34 (51) |
| <50% | 30 (45) |
| Not determined | 3 (6) |
| Cytogenetics (%) |  |
| normal | 31 (46) |
| complex | 9 (13) |
| t8;21 | 3 (4) |
| inv16 | 2 (3) |
| Other | 22 (36) |
| Mutations (% tested positive) |  |
| FLT3-ITD | 26 (39) |
| NPM1 | 19 (28) |
| IDH1 | 2 (3) |
| IDH2 | 9 (13) |
| RUNX1-RUNX1T1 | 3 (4) |
| CEBPA | 7 (10) |
| KMT2A-PTD | 7 (10) |
| PML-RARA | 0 (0) |
| CBFb-MYH11 | 4 (6) |
